# Hepatitis B virus reactivation in cancer patients with positive Hepatitis B surface antigen undergoing PD-1 inhibition

**DOI:** 10.1186/s40425-019-0808-5

**Published:** 2019-11-21

**Authors:** Xuanye Zhang, Yixin Zhou, Chen Chen, Wenfeng Fang, Xiuyu Cai, Xiaoshi Zhang, Ming Zhao, Bei Zhang, Wenqi Jiang, Zuan Lin, Yuxiang Ma, Yunpeng Yang, Yan Huang, Hongyun Zhao, Ruihua Xu, Shaodong Hong, Li Zhang

**Affiliations:** 1 State Key Laboratory of Oncology in South China, Guangzhou, China; 2 Collaborative Innovation Center for Cancer Medicine, Guangzhou, China; 3 Department of Medical Oncology, Sun Yat-sen University Cancer Center, 651 Dongfeng East Road, Guangzhou, 510060 China; 4 Department of VIP region, Sun Yat-sen University Cancer Center, Guangzhou, China; 5 Department of Radiotherapy, Sun Yat-sen University Cancer Center, Guangzhou, China; 6 Biotherapy Center, Sun Yat-sen University Cancer Center, Guangzhou, China; 7 Department of Minimally Invasive Interventional Radiology, Center of Medical Imaging and Interventional Radiology, Sun Yat-sen University Cancer Center, Guangzhou, China; 8 Department of Clinical Research, Sun Yat-sen University Cancer Center, Guangzhou, China

**Keywords:** PD-1, PD-L1, Immunotherapy, Checkpoint, Cancer, Hepatitis B virus, Reactivation, Safety

## Abstract

**Background:**

Hepatitis B virus (HBV) reactivation is a serious complication in patients with cancers and HBV infection undergoing immunosuppressant treatment or chemotherapy. However, the safety of anti-programmed cell death (PD) -1 and anti-programmed cell death-ligand 1 (PD-L1) therapy in these patients is unknown because they were excluded from clinical trials of immunotherapy.

**Methods:**

This retrospective cohort study involved consecutive hepatitis B surface antigen (HBsAg) -positive cancer patients who were referred to Sun Yat-sen University Cancer Center and received an anti-PD-1/PD-L1 antibody between January 1, 2015 and July 31, 2018. The primary end point was the rate of the occurrence of HBV reactivation.

**Results:**

In total, 114 eligible patients were included, among whom 90 (79%) were male, and the median (range) age was 46 (16–76) years. Six patients (5.3%) developed HBV reactivation, occurring at a median of 18 weeks (range, 3–35 weeks) from the commencement of immunotherapy. Among these patients, all of them had undetectable baseline HBV DNA; one had prophylactic antiviral therapy while five did not; four were positive for Hepatitis B e antigen while the other two were negative. At reactivation, the median HBV DNA level was 3.89 × 10^4^ IU/mL (range, 1.80 × 10^3^–6.00 × 10^7^ IU/mL); five had HBV-related hepatitis and one exhibited increasing HBV DNA level without alanine transaminase elevation. No HBV-related fatal events occurred. The lack of antiviral prophylaxis was the only significant risk factor for HBV reactivation (odds ratio, 17.50 [95% CI, 1.95–157.07], *P* = .004).

**Conclusions:**

HBV reactivation occurs in a subset of HBsAg-positive cancer patients undergoing anti-PD-1 or anti-PD-L1 immunotherapy. Regular monitoring of HBV DNA and antiviral prophylaxis are advised to prevent this potentially fatal complication.

## Background

Anti-programmed cell death (PD) -1 and anti-programmed cell death-ligand 1 (PD-L1) blockade have revolutionized the treatment of cancers, with regulatory approval for patients with various cancer types [1]. The indications of anti-PD-(L)1 immunotherapy continue to expand at a rapid pace. Therefore, an increasing number of patients will be exposed to the toxicities of these agents, which are related to the mechanism of action that is distinct from chemotherapy and targeted therapy [2]. In most clinical trials of immunotherapy, patients with pre-existing virus infection, such as hepatitis B virus (HBV), hepatitis C virus (HCV) or human immunodeficiency virus (HIV) infection, are excluded. Therefore, the safety of immune checkpoint inhibitor in these patients remains unknown.

The challenge is that more than 350 million people have chronic HBV infection worldwide, and about 75% of them are from Southeast Asia and the Western Pacific regions [3, 4]. HBV reactivation induced by immunosuppressive agents or cytotoxic chemotherapy is a well-recognized complication in cancer patients with pre-existing HBV infection. HBV reactivation could lead to a variety of clinical manifestations, ranging from asymptomatic hepatitis to fatal liver damage [5]. Therefore, antiviral prophylaxis is now routinely prescribed for patients with positive HBV surface antigen (HBsAg) who receive immunosuppressive agents such as rituximab [6].

Unfortunately, there are limited published data describing the safety of anti-PD-(L)1 antibody for patients with advanced cancers and HBV infection. Several case reports have demonstrated that HBV reactivation does occur in some patients with resolved HBV infection during anti-PD-1 therapy [7–9]. However, the rate of HBV reactivation and potential risk factors are not defined. In this retrospective cohort study, we aimed to evaluate the rate of HBV reactivation in a large cohort of HBsAg-positive cancer patients undergoing anti-PD-1 or anti-PD-L1 therapy.

## Patients and methods

### Study design and participants

We performed a retrospective cohort study of anti–PD-1 or anti–PD-L1 therapy in cancer patients who were seropositive for HBsAg. This study involved consecutive patients referred to Sun Yat-sen University Cancer Center in Guangzhou, China, between January 1, 2015 and July 31, 2018. A total of 1310 cancer patients were screened for eligibility. The inclusion criteria were as follows: (1) pathologically diagnosed with malignant tumor; (2) received at least one cycle of anti–PD-1 or anti–PD-L1 therapy; (3) had been tested for hepatitis virus infection and were seropositive for HBsAg; (4) with HBV DNA and liver function monitored regularly during immunotherapy and the follow-up period, according to the treating physician. Patients were excluded if they had other positive viral markers including IgM antibody to hepatitis A virus (HAV), antibody to HCV, IgG antibody to hepatitis D virus (HDV), IgM antibody to hepatitis E virus (HEV), or antibody to HIV. The study protocol conforms to the ethical guidelines of the 1975 Declaration of Helsinki as reflected in a priori approval by the Sun Yat-Sen University Cancer Center Institutional Review Board. Informed consent was obtained from all patients before conducting the treatment.

The primary study end point was HBV reactivation, which was defined according to the American Association for the Study of Liver Diseases (AASLD) 2018 hepatitis B guidance: [6] (1) a ≥ 2 log (100-fold) increase in HBV DNA compared to the baseline level, (2) HBV DNA ≥ 3 log (1000) IU/mL in a patient with previously undetectable level, or (3) HBV DNA ≥ 4 log (10,000) IU/mL if the baseline level is not available. Hepatitis was categorized into HBV-related hepatitis, cytotoxic drug-related hepatitis, hepatitis attributed to hepatic lesion progression, and immune-related hepatitis, according to the judgement of the treating physician and the corresponding authors, based on clinical manifestations, laboratory tests, and imaging. Hepatitis was defined as a three-fold or greater increase in serum ALT level that exceeded the reference range (58 U/L) or an absolute increase of ALT to more than 100 U/L. HBV-related hepatitis was defined as hepatitis accompanying or following HBV reactivation in absence of acute infection with other hepatitis viruses or systemic disease [10, 11]. Antiviral prophylaxis was defined as anti-HBV treatment administered before and during anti-PD-1 therapy. The severity of hepatitis was graded according to the National Cancer Institute Common Toxicity Criteria (CTCAE) version 4.0.

Serological markers for HBV infection (including HBsAg, anti-HBs antibody, anti-HBc antibody, HBeAg, and anti-HBe antibody) were routinely tested in our center. Serum HBV DNA was monitored every 1 to 3 months according to the decision of the treating physician and was measured by real-time viral polymerase chain reaction (PCR) in our center using an ABI 7900 real-time thermo-cycler (ABI 7900; Applied Biosystems, Foster City, CA, USA) with a lower limit of 10 IU/mL.

### Statistical analysis

Data were extracted from the patients’ medical records. Qualitative variables were reported as the frequency (percentage), and quantitative variables were reported as the median (range). The primary endpoint of this study was the rate of the occurrence of HBV reactivation. Secondary endpoints included the risk factors for HBV reactivation and hepatitis of any etiology. Qualitative variables were compared using the Pearson *χ*2 or Fisher exact test, where appropriate. Bivariable analyses were performed to assess the association between potential factors and HBV reactivation or hepatitis of any etiology, including age, gender, antiviral prophylaxis, performance status, history of alcoholism, liver involvement, liver cirrhosis, HBeAg status, baseline HBV DNA level, treatment modality (anti-PD-1 monotherapy vs. combination therapy), and the use of concurrent steroids. A 2-tailed *P* value of ≤0.05 defined statistical significance. All statistical analyses were performed using SPSS version 22.0 (IBM, Armonk, NY, USA).

## Results

### Patients

**Fig. 1:**
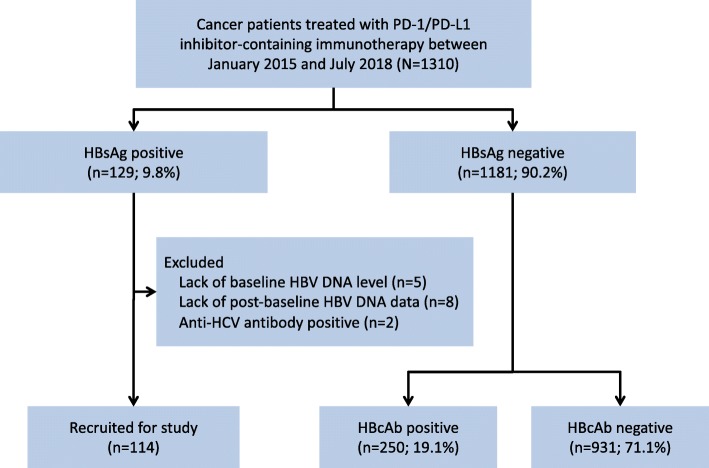
Flow chart depicting patient deposition. PD-1, programmed cell death 1; PD-L1, programmed cell death-ligand 1; HBsAg, hepatitis B surface antigen; HBV, hepatitis B virus; anti-HCV, antibody to the hepatitis C virus; HBcAb, hepatitis B core antibody

Of the 1310 patients referred to Sun Yat-sen University Cancer Center over the study period, 129 were seropositive for HBsAg. Fifteen patients were excluded: 5 lacked baseline HBV DNA level, 8 lacked post-baseline HBV DNA data, 2 were positive for Anti-HCV antibody. No other patients were excluded for co-infection with HAV, HDV, HEV, or HIV. Ultimately, 114 eligible patients were included in the study (Fig. 1). The patient characteristics are summarized in Table 1. Patients were predominantly male (*n* = 90, 78.9%) and the median age was 46 years (range, 16–76). The main tumor types were nasopharyngeal carcinoma (NPC; *n* = 35, 24.6%), hepatocellular carcinoma (HCC; *n* = 28, 24.6%), melanoma (*n* = 14, 12.3%) and non-small cell lung carcinoma (NSCLC; *n* = 13, 11.4%). Eighty-three patients (72.8%) received anti-PD-1/PD-L1 monotherapy, whereas 31 (27.2%) were treated with combination therapy. The median duration of anti-PD-1/PD-L1 treatment was 10 weeks (range, 1–102 weeks). Eighty-five patients (74.6%) were on antiviral prophylaxis prior to anti-PD-1/PD-L1 therapy, and the most commonly used agent was entecavir (*n* = 68, 59.6%). At baseline, 35 patients (30.7%) had detectable HBV DNA with a median titre of 4.82 × 10^2^ IU/mL (range, 30.1–2.48 × 10^5^ IU/mL). Among 35 patients with detectable HBV DNA, 85.7% (*n* = 30) received antiviral prophylaxis; while among 79 patients with undetectable HBV DNA, only 69.6% (*n* = 55) were on antiviral prophylaxis.

**Table 1:** Baseline characteristics of the included patients (*n* = 114) and the primary outcome

	No. of patients (%)	No. of HBV reactivation events (%)	OR (95% CI)	*P* value^a^
Age				
<40	26 (22.8)	2 (7.7)	1.75 (0.30–10.14)	0.895
≥40	88 (77.2)	4 (4.5)	1	
Median age (range), years	46 (16–76)			
Gender				
Male	90 (78.9)	5 (5.6)	1.35 (0.30–10.14)	1.000
Female	24 (21.1)	1 (4.2)	1	
Cancer type				
Hepatocellular carcinoma	28 (24.6)	1 (3.6)	0.54 (0.060–4.84)	0.667
Lymphoma	8 (7.0)	0 (0)	0.87 (0.040–15.49)	
Others^b^	78 (68.4)	5 (6.4)	1	
ECOG performance status				
≤1	94 (82.5)	6 (6.4)	3.01 (0.16–55.63)	0.542
>1	20 (17.5)	0 (0)	1	
History of alcoholism				
Yes	17 (14.9)	0 (0)	0.40 (0.022–7.47)	0.589
No	97 (85.1)	6 (6.2)	1	
Liver involvement^c^				
Yes	73 (64.0)	3 (4.1)	0.54 (0.10–2.82)	0.765
No	41 (36.0)	3 (7.3)	1	
Liver cirrhosis				
Yes	33 (28.9)	1 (3.0)	0.48 (0.053–4.23)	0.827
No	81 (81.1)	5 (6.2)	1	
HBeAg status				
Seropositive^d^	12 (10.5)	2 (16.7)	6.25 (0.99–39.50)	0.086
Seronegative	102 (89.5)	4 (3.9)	1	
Baseline HBV DNA level				
Detectable^e^	35 (30.7)	0 (0)	0.16 (0.0087–2.91)	0.222
Undetectable	79 (69.3)	6 (7.6)	1	
Median baseline HBV DNA (range), IU/mL	0 (0–2.48 × 10^5^)			
Previous lines of therapy				
<2	70 (61.4)	3 (4.3)	0.61 (0.12–3.18)	0.874
≥2	44 (38.6)	3 (6.8)	1	
Treatment modality				
PD-1/PD-L1 inhibitor^f^ monotherapy	83 (72.8)	6 (7.2)	5.28 (0.29–96.62)	0.286
Combination therapy^g^	31 (27.2)	0 (0)	1	
Concurrent steroids^h^				
Yes	14 (12.3)	1 (7.1)	1.46 (0.15–13.51)	0.553
No	100 (87.7)	5 (5.0)	1	
Antiviral prophylaxis				
No	29 (25.4)	5 (17.2)	17.50 (1.95–157.07)	0.004
Yes^i^	85 (74.6)	1 (1.2)	1	
Antiviral prophylaxis agents				
Entecavir	68 (59.6)	1	NC	NC
Lamivudine	10 (8.8)	0	NC	
Tenofovir	5 (4.4)	0	NC	
Telbivudine	1 (0.9)	0	NC	
Adefovir	1 (0.9)	0	NC	
Nil	29 (25.4)	5	NC	

^a^Calculated using the χ2 test except for history of alcoholism, HBeAg status and concurrent steroids which were calculated using the Fisher exact test

^b^Including nasopharyngeal carcinoma (*n* = 35), melanoma (*n* = 14), non-small cell lung cancer (*n* = 13), colorectal cancer (*n* = 4), gastric cancer (*n* = 2), esophageal cancer (*n* = 2), head and neck squamous cancer (*n* = 1), urothelial carcinoma (*n* = 1), breast cancer (n = 1), soft tissue sarcoma (*n* = 1), ovarian cancer (*n* = 1), neuroendocrine carcinoma of the skin (Merkle cell carcinoma, *n* = 1) and carcinoma of unknown primary origin (*n* = 2)

^c^Including primary liver cancer and liver metastasis

^d^One did not received antiviral prophylaxis; 10 received entecavir and 1 received tenofovir as antiviral prophylaxis

^e^HBV DNA ≥ 10 IU/mL

^f^Including pembrolizumab, nivolumab, toripalimab, camrelizumab, sintilimab, atezolizumab

^g^Including PD-1/PD-L1 inhibitor plus chemotherapy (*n* = 22), targeted agent (osimertinib [n = 1], bevacizumab [n = 1], regorafenib [n = 1], apatinib [n = 1], sunitinib [n = 1], nimotuzumab [n = 2], cetuximab [n = 1]) and ipilimumab (n = 2)

^h^Systemic steroids for any reason during immunotherapy, including premedication, treatment for high intracranial pressure and treatment for immune-related adverse events

^i^Including entecavir (*n* = 68), lamivudine (*n* = 10), tenofovir (*n* = 5), telbivudine (n = 1) and adefovir (n = 1)

Abbreviations: *HBV* hepatitis B virus, *OR* odds ratio, *CI* confidence interval, *ECOG* Eastern Cooperative Oncology Group, *HBeAg* Hepatitis B e antigen, *HBV* hepatitis B virus, *PD*-1, programmed cell death protein-1, *PD-L*1 programmed cell death-ligand 1, *NC* not computable

### HBV reactivation and hepatitis

**Fig. 2:**
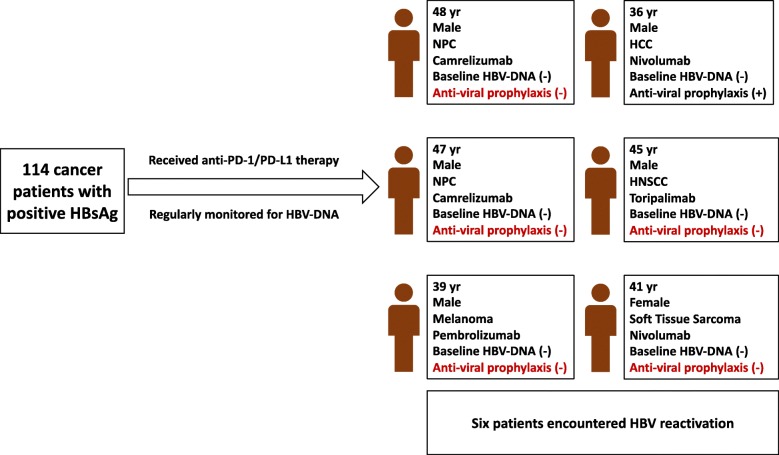
Characteristics of the six patients suffering from HBV reactivation. HBsAg, hepatitis B surface antigen; HBV, hepatitis B virus; PD-1, programmed cell death 1; PD-L1, programmed cell death-ligand 1; NPC, nasopharyngeal carcinoma; HCC, hepatocellular carcinoma; HNSCC, head and neck squamous cancer

Six (5.3%) of 114 patients developed HBV reactivation with a median onset of 18 weeks (range, 3–35 weeks) after anti-PD-1/PD-L1 therapy. Details of the six patients with HBV reactivation are listed in Table 2 and Fig. 2. The underlying malignancies of these patients were NPC (*n* = 2), melanoma (*n* = 1), HCC (n = 1), head and neck squamous cell cancer (n = 1) and soft tissue sarcoma (n = 1). All the six patients were treated with anti-PD-1 antibody single agent. Five episodes of HBV reactivation occurred during immunotherapy; while the remaining one case occurred six weeks after immunotherapy was discontinued.

**Table 2:** Details of the 6 Patients with HBV reactivation

Patients Characteristics				Baseline			At reactivation						
Patient	Age (years)	Gender	Cancer type	Anti-tumor therapy	HBV DNA (IU/mL)	Antiviral prophylaxis	Weeks from start of immunotherapy	HBV DNA (IU/mL)	Peak ALT (U/L)	Anti-PD-1/PD-L1 therapy disruption	Antiviral treatment	Time for achieving HBV-DNA undetectable (weeks)	Time for ALT recovery (weeks)
1	48	M	NPC	Camrelizumab	Undetectable	Nil	3	7.81 × 10^3^	191.4	Delayed	Entecavir	1	2
2	47	M	NPC	Camrelizumab	Undetectable	Nil	16	6.98 × 10^4^	203.0	Delayed	Entecavir	4	4
3	39	M	Melanoma	Pembrolizumab	Undetectable	Nil	28	2.10 × 10^3^	27.6	No	Nil	5	NA
4	36	M	HCC	Nivolumab	Undetectable	Entecavir	12	1.80 × 10^3^	298	Discontinued	Entecavir plus tenofovir	1	3
5	45	M	HNSCC	Toripalimab	Undetectable	Nil	35	4.04 × 10^6^	281.2	Delay	Entecavir	3	6
6^a^	41	F	Soft Tissue Sarcoma	Nivolumab	Undetectable	Nil	20	6.00 × 10^7^	465.1	NA	Entecavir	8	4

^a^HBV reactivation in this patient occurred 6 weeks after immunotherapy was discontinued; other HBV reactivation occurred during anti-PD-1/PD-L1 thearpy

Abbreviations: *M* male, *F* female, *HBV* hepatitis B virus, *NPC* nasopharyngeal carcinoma, *HCC* hepatocellular carcinoma, *HNSCC* head and neck squamous cell cancer, *ALT* alanine aminotransferase, *NA* not applicable

All the six patients had undetectable baseline HBV DNA (< 10 IU/mL). At reactivation, the median HBV DNA level was 3.89 × 10^4^ IU/mL (range, 1.80 × 10^3^–6.00 × 10^7^ IU/mL). Five patients were diagnosed with HBV-related hepatitis with a median peak ALT of 281.2 U/L (range, 191.4–465.1 U/L); one patient only exhibited a brief increase in HBV DNA level without ALT elevation.

One patient received entecavir as antiviral prophylaxis before the commencement of immunotherapy, while the remaining five did not receive antiviral prophylaxis. Among the five patients without antiviral prophylaxis, four were given entecavir after the occurrence of reactivation and had resolution of hepatitis thereafter; one did not receive salvage antiviral treatment but the HBV DNA spontaneously turned undetectable 6 weeks later without ALT elevation. For the one with prophylactic entecavir, antiviral treatment was modified to entecavir plus tenofovir at reactivation.

Four patients experienced immunotherapy disruption due to HBV reactivation, including one case of immunotherapy discontinuation and three cases of treatment delay. No HBV-related fatal events occurred during the study period. For the six patients with HBV reactivation, all achieved undetectable HBV DNA levels after a median of 3.5 weeks (range, 1–8 weeks). For the five patients with HBV-related hepatitis, liver enzymes turned normal after a median of 3 weeks (range, 2–6 weeks).

All grade hepatitis occurred in 35 (30.7%) patients, including five (4.4%) cases of HBV-related hepatitis and 15 (13.2%) cases of immune-related hepatitis. The causes of hepatotoxicity in the other cases were disease progression in hepatic lesion (*n* = 9) and cytotoxic drugs (*n* = 6). Ten (8.8%) patients experienced grade 3/4 hepatitis (four HBV-related hepatitis; four immune-related hepatitis; one cytotoxic drug-related hepatitis and one attributed to hepatic lesion progression). Among the 35 patients with all grade hepatitis, 20 of them had a complete recovery of liver enzymes after a median of 3.5 weeks (range, 1–17 weeks).

Six patients received steroids for immune-related adverse events (irAEs) during anti-PD-1/PD-L1 therapy (Additional file 1: Table S1), including one with grade 2 immune-related hepatitis, four with grade 3 immune-related hepatitis, and one with grade 2 immune-related pneumonitis. None of these patients had HBV reactivation during or after steroid treatment.

Among the 35 patients with hepatitis, ten had immunotherapy delay and one had discontinuation of anti-PD-1 treatment (Table 3). The average duration of treatment delay to allow recovery for the ten patients was 43 days (range, 14–121 days). After re-challenge with anti-PD-1/PD-L1 treatment, neither further episodes of HBV reactivation nor worsening of liver function occurred.

**Table 3:** Efficacy of antiviral prophylaxis in HBsAg-positive patients

Events	No. (%) of patients			Difference between groups, % (95% CI)	OR (95% CI)	*P* value^a^
	Total (*n* = 114)	Patients without antiviral prophylaxis (*n* = 29)	Patients with antiviral prophylaxis (*n* = 85)			
Hepatitis						
All grades	35 (30.7)	8 (27.6)	27 (31.8)	4.2 (−16.01–20.83)	0.82 (0.32–2.08)	0.674
Grade 3/4	10 (8.8)	4 (13.8)	6 (7.1)	6.7 (−4.50–23.89)	2.10 (0.55–8.07)	0.467
HBV reactivation	6 (5.3)	5 (17.2)	1 (1.2)	16.0 (5.05–33.33)	17.50 (1.95–157.07)	0.004
HBV-related hepatitis	5 (4.4)	4 (13.8)	1 (1.2)	12.6 (2.80–29.40)	13.44 (1.44–152.79)	0.019
Immunotherapy disruption^b^	11 (9.6)	4 (13.8)	7 (8.2)	5.6 (−5.78–22.88)	1.78 (0.48–6.60)	0.609

^a^Determined using the χ2 test

^b^Included ten cases of immunotherapy delay and one case of discontinuation

Abbreviations: *HBsAg* hepatitis B surface antigen, *HBV* hepatitis B virus, *OR* odds ratio, *CI* confidence interval

### Efficacy of antiviral prophylaxis in HBsAg-positive patients

Patients with antiviral prophylaxis had significant lower HBV reactivation rate than those without antiviral prophylaxis (1.2% vs. 17.2%, *P* = .004). The incidence of HBV-related hepatitis also was significantly lower in the prophylaxis group (1.2% vs. 13.8%, *P* = .019) (Table 3). No significant differences were found in all grade hepatitis, grade 3/4 hepatitis, or immunotherapy disruption between the two groups.

### Factors associated with HBV reactivation and hepatitis

As shown in Table 1, the lack of antiviral prophylaxis was the only significant risk factor for HBV reactivation (Odds ratio [OR], 17.50 [95% CI, 1.95–157.07]; *P* = .004). Patients who were seropositive for HBeAg appeared to have increased risk of HBV reactivation, though not statistically significant (OR, 6.25 [95% CI, 0.99–39.50]; *P* = .086). Neither baseline HBV DNA level nor treatment modality was associated with HBV reactivation.

Patients with HCC had higher risk of any-grade hepatitis than those with other cancer type (OR, 2.52 [95% CI, 1.04–6.12]; *P* = .038). No other significant risk factors for all grade hepatitis, grade 3/4 hepatitis, and immune-related hepatitis were identified in this study (Additional file 2: Table S2 and Additional file 3: Table S3).

## Discussion

To our best knowledge, this is the first systematic analysis of the incidence of HBV reactivation in a large cohort of HbsAg-positive patients undergoing anti-PD-1/PD-L1 therapy. The study showed that HBV reactivation occurs in 5.3% of the patients and the lack of prophylactic antiviral therapy was the most important risk factor (OR 17.50). These findings are of particular clinical relevance due to the large population base with chronic HBV infection and their exclusion from clinical trials of immunotherapy. With the increasing use of immune checkpoint inhibitor for cancer patients, HBV reactivation will pose an increasing clinical challenge, especially in endemic areas.

Thus far, only three isolated incidents of HBV reactivation in patients with resolved HBV infection (HBsAg-negative and HBcAb-positive) who received anti-PD-1 therapy have been reported [7–9]. In a case series enrolling 14 patients with advanced cancers and hepatitis B undergoing anti-PD-(L)1 therapy, none developed hepatitis or had a ≥ one log increase in the viral load [12]. There also are very limited data regarding virus reactivation in HBsAg-positive patients from prospective studies. In the CheckMate 040 study, 15 HBV-infected patients with HCC were treated with nivolumab and none of them had HBV reactivation [13]. These patients were required to be receiving effective antiviral therapy and have a viral load of less than 100 IU/mL at screening. However, these patients were only regularly monitored for HBsAg but not HBV DNA. In the KEYNOTE-224 study, 22 patients with hepatitis B and advanced HCC were treated with pembrolizumab [14]. These patients also were required to undergo antiviral therapy and have a viral load of less than 100 IU/mL before receiving pembrolizumab. There were no cases of HBV flares (defined as elevations of ALT and AST to > 5 × ULN and/or > 3× baseline); but the rate of reactivation was not reported. Unfortunately, all these studies provided no information on the serologic classification. Whether these patients were in active or resolved infection was unclear. Also, the sample sizes are too small to reach robust conclusions. Therefore, these data do not allow full evaluation of the incidence of and risk factors for HBV reactivation as well as the necessity of antiviral prophylaxis in HBsAg-positive patients receiving immune checkpoint inhibitor.

The mechanism of HBV reactivation induced by anti-PD-1/PD-L1 therapy is unclear. The PD-1/PD-L1 axis is a critical pathway for maintaining immune homeostasis [15]. Apart from being involved in cancer immune evasion, [16] this pathway also plays a role in the course of hepatitis virus infection [17, 18]. On one hand, HBV-specific CD8+ T cells could express PD-1 molecule in chronic HBV infection and their antiviral function could be partially restored by blocking the PD-1/PD-L1 engagement [19, 20]. On the other hand, PD-1 is an important immunosuppressive mediator that helps prevent overwhelming liver damage. Therefore, blocking the PD-1/PD-L1 axis may lead to the destruction of hepatocytes and the release of previously latent virus into circulation [21, 22]. Furthermore, PD-1 may suppress the proliferation of T regulatory cells (Tregs). The blockade of PD-1 may promote the proliferation of Tregs that leads to increased immunosuppression, hence the reactivation of HBV [23, 24]. In line with these inconsistent hypotheses, the only clinical trial with anti-PD-1 antibody for non-cancer patients with viral hepatitis showed that even though some patients have persistent suppression of HCV replication, only 5 of 42 patients (12%) met the primary endpoint of a ≥ 0.5 log reduction in HCV RNA [25]. Although this study did not provide information on the occurrence of increased HCV load, it could not rule out the possibility of virus reactivation in patients undergoing anti-PD-1 therapy. More basic research will be needed to reveal the underlying mechanisms of hepatitis virus reactivation due to anti-PD-1 therapy.

While consensus on the needs for antiviral prophylaxis and close monitoring of HBV reactivation is established in patients who are HBsAg-positive and receiving immunosuppressive agents or chemotherapy, our knowledge about the safety of immune checkpoint inhibitor for these patients are scarce [5]. This could be reflected from the fact that 29 patients (25%) in our study did not receive prophylactic antiviral therapy. Among the 6 patients with HBV reactivation, 5 did not received prophylactic antiviral treatment. The risk of HBV reactivation was 16 times higher in patients without prophylaxis than those with prophylaxis (17.2% vs. 1.2%; OR 17.50; *P* = .004). Also, the lack of antiviral prophylaxis was significantly associated with higher risk of HBV-related hepatitis (13.8% vs. 1.2%; OR 13.44; *P* = .019). These results indicate that HBsAg-positive patients should have effective antiviral treatment before and during anti-PD-1 therapy. Notably, one patient still developed HBV reactivation despite entecavir prophylaxis, probably because of the development of antiviral drug resistance [26]. This case implies that close monitoring of HBV status is also needed for patients receiving antiviral prophylaxis.

Currently, some recognized risk factors for HBV reactivation include male sex, older age, presence of cirrhosis, and type of disease needing immunosuppression, high baseline HBV-DNA level and HBeAg positivity [5]. However, we failed to identify any one of these factors that significantly contributed to HBV reactivation in this study. Intriguingly, all the 6 cases of reactivation occurred in those with undetectable baseline HBV DNA. This is probably because some physicians think that antiviral prophylaxis could be safely omitted in patients with undetectable baseline HBV DNA. This also implies that anti-PD-1 therapy is quite safe in patients with detectable baseline HBV DNA. We also found that patients with positive HBeAg tended to have higher risk of reactivation, though not statistically significant (20% vs. 3.8%; OR 6.25; *P* = .086). HBeAg positivity indicates that HBV is under active replication and there is a higher probability of virus reactivation.

Another relevant finding is that one case of virus reactivation occurred 6 weeks after immunotherapy was ended, implying that the effect of PD-1 blockade could persist beyond treatment period. Currently, it is recommended that antiviral therapy should be continued for at least 6 months after the last dose of immunosuppressive agents or chemotherapy. However, the optimal duration of antiviral therapy for patients undergoing PD-1 inhibitor treatment is unclear. It is also not sure which antiviral agent is the most appropriate in terms of efficacy and cost trade-off.

Interestingly, the rate of hepatitis and immune-related hepatitis is higher than previously reported for anti-PD-1 single agent or combination therapy [27]. This raises the possibility that patients who are HBsAg-positive may be at higher risk of having concurrent immune related hepatitis, which requires greater vigilance and further study.

A limitation of this study is that the interval of HBV DNA monitoring varied within and among patients. Therefore, the rate and median time of the episode of HBV reactivation might be underestimated. However, with this retrospective nature, we were able to analyze the risk of reactivation in patients with vs. without antiviral prophylaxis. This also enabled us to analyze the safety of anti-PD-1 therapy in those with high baseline HBV DNA level. Other limitations included the relatively small sample size and numbers of outcomes analyzed. For example, we could not explore the association between HBV status, occurrence of HBV reactivation, or use of prophylaxis and response to immunotherapy. Nevertheless, this is currently the largest cohort study with HBsAg-positive patients treated with PD-1 inhibitors. The possibility of HBV reactivation, though relatively low, should be considered seriously for these patients. Furthermore, patients were recruited from endemic area whose HBV genotypes are different from other population. Whether these results could be applied elsewhere remains to be elucidated. Additionally, we did not evaluate the HBV reactivation events in patients with resolved HBV infection. This is due to the fact that most of the patients with resolved HBV infection did not receive regular HBsAg status or HBV DNA monitoring during anti-tumor treatment in the real-world setting. Considering these limitations, further studies with extended sample size are strongly encouraged to identify risk factors for reactivation and to optimize the monitoring, prevention and management of HBV reactivation in patients who are HBV-infected and undergoing immunotherapy.

**Fig. 3:**
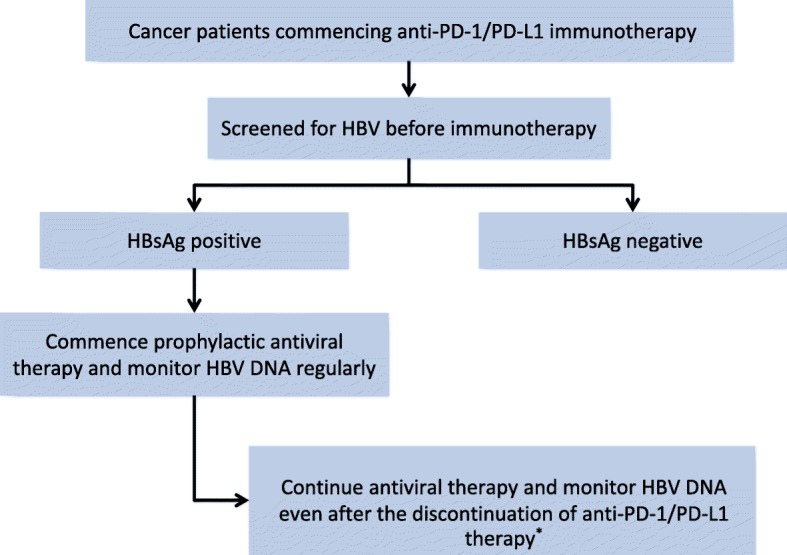
Proposed management strategy for hepatitis B surface antigen (HBsAg)-positive patients starting anti-PD-1-antibody-containing immunotherapy. (^*^) The optimal duration of prophylactic antiviral therapy after the discontinuation of anti-PD-1 therapy remains to be determined. Anti-PD-1, anti-programmed cell death 1; PD-L1, programmed cell death-ligand 1; HBsAg, hepatitis B surface antigen; HBV, hepatitis B virus

In summary, HBsAg positivity should not be a contraindication for immune checkpoint inhibitor treatment. However, HBV reactivation does occur in a small subset of patients who are seropositive for HBsAg. Therefore, universal screening with serologic tests for hepatitis B should be performed before anti-PD-1/PD-L1 therapy. For those who are seropositive for HBsAg, initiation of prophylactic antiviral treatment is recommended irrespective of baseline HBV DNA level, as depicted in Fig. 3.

### Financial support

This study was funded by grants 2016YFC0905500 and 2016YFC0905503 from the National Key R&D Program of China; 81,972,898, 81,602,005, 81,702,283, 81,872,499, and 81,602,011 from the National Natural Science Funds of China; 16zxyc04 from the Outstanding Young Talents Program of Sun Yat-sen University Cancer Center; 17ykpy81 from the Central Basic Scientific Research Fund for Colleges-Young Teacher Training Program of Sun Yat-sen University; 2017B020227001 from the Science and Technology Program of Guangdong Province. The funding sources had no role in the design and conduct of the study; collection, management, analysis, and interpretation of the data; preparation, review, or approval of the manuscript; and decision to submit the manuscript for publication.

## Supplementary information

**Additional file 1: Table S1.** Details of 6 patients receiving steroids for irAE.

**Additional file 2: Table S2.** Analysis of factors associated with any grade hepatitis and grade 3/4 hepatitis.

**Additional file 3: Table S3.** Analysis of factors associated with immune-related hepatitis.

## Data Availability

All data generated or analyzed during this study are included in this published article and its supplementary information files.

## Abbreviations

AASLD: American Association for the Study of Liver Diseases
ALT: alanine aminotransferase
AST: aspartate aminotransferase
CTCAE: National Cancer Institute Common Toxicity Criteria
HAV: hepatitis A virus
HBsAg: HBV surface antigen
HBV: Hepatitis B virus
HCC: hepatocellular carcinoma
HCV: hepatitis C virus
HDV: hepatitis D virus
HIV: immunodeficiency virus
IHEV: hepatitis E virus
irAEs: immune-related adverse events
NPC: nasopharyngeal carcinoma
NSCLC: non-small cell lung carcinoma
OR: Odds ratio
PD-1: programmed cell death 1
PD-L1: programmed cell death 1 ligand 1
