# Supplementary material for: Hepatitis B virus reactivation in cancer patients with positive Hepatitis B surface antigen undergoing PD-1 inhibition
Source: J Immunother Cancer. 2019 Nov 21;7:322. doi: 10.1186/s40425-019-0808-5 (PMC6873745; doi:10.1186/s40425-019-0808-5)
Supplement: Supplementary file 1 — Additional file 1: Table S1. Details of 6 patients receiving steroids for irAE. [file 40425_2019_808_MOESM1_ESM.docx]

**Supplementary Table 1. Details of 6 patients receiving steroids for irAE**

| Patient | Age (year) | Gender | Cancer type | Anti-tumor therapy | irAE | Grade | Peak ALT (U/L) | Peak TBil (IU/L) | Dosage and duration of initial steroid | Duration of steroid tapering (weeks) | Outcome |
| --- | --- | --- | --- | --- | --- | --- | --- | --- | --- | --- | --- |
| 1 | 34 | F | HCC | Nivolumab+ regorafenib | Hepatitis | 3 | 231.0 | 14.4 | Prednisone 1 mg/kg for 7 days | 4 | Recovery and continued immunotherapy |
| 2 | 53 | M | HCC | Nivolumab | Hepatitis | 3 | 304.0 | 46.5 | Methylprednisone 500 mg for 3 days, then prednisone 2 mg/kg for 6 days | 6 | Recovery and continued immunotherapy |
| 3 | 41 | F | NSCLC | Camrelizumab | Hepatitis | 3 | 530.3 | 6.5 | Prednisone 2 mg/kg for 7 days | 4 | Recovery and continued immunotherapy |
| 4 | 30 | M | Melanoma | Pembrolizumab | Hepatitis | 3 | 230.5 | 7.6 | Prednisone 1 mg/kg for 15 days | 4 | Recovery and continued immunotherapy |
| 5 | 52 | M | Melanoma | Nivolumab | Hepatitis | 2 | 198.0 | 16.8 | Prednisone 0.5 mg/kg for 8 days | 3 | Recovery and continued immunotherapy |
| 6 | 45 | M | NPC | Camrelizumab | Pneumonitis | 2 | NA | NA | Methylprednisone 500 mg for 3 days, then prednisone 1 mg/kg for 14 days | 6 | Recovery and continued immunotherapy |

Abbreviations: irAE, immune-related adverse event; F, female; M, male; HCC, hepatocellular carcinoma; NSCLC, non-small cell lung carcinoma; NPC, nasopharyngeal carcinoma; ALT, alanine aminotransferase; TBil, total bilirubin; NA, not applicable.
