# Supplementary material for: Hepatitis B virus reactivation in cancer patients with positive Hepatitis B surface antigen undergoing PD-1 inhibition
Source: J Immunother Cancer. 2019 Nov 21;7:322. doi: 10.1186/s40425-019-0808-5 (PMC6873745; doi:10.1186/s40425-019-0808-5)
Supplement: Supplementary file 2 — Additional file 2: Table S2. Analysis of factors associated with any grade hepatitis and grade 3/4 hepatitis. [file 40425_2019_808_MOESM2_ESM.docx]

**Supplementary Table 2．Analysis of factors associated with any grade hepatitis and grade 3/4 hepatitis**

|  | No. of patients (%) | Any grade hepatitis | | |  | Grade 3/4 hepatitis | | |
| --- | --- | --- | --- | --- | --- | --- | --- | --- |
|  |  | No. of events (%) | OR (95%CI) | P value^a^ |  | No. of events (%) | OR (95%CI) | P value^b^ |
| Age |  |  |  |  |  |  |  |  |
| < 40 | 26 (22.8) | 10 (38.5) | 1.58 (0.63-3.94) | 0.392 |  | 3 (11.5) | 1.51 (0.36-6.30) | 0.863 |
| ≥ 40 | 88 (77.2) | 25 (28.4) | 1 |  |  | 7 (8.0) | 1 |  |
| Gender |  |  |  |  |  |  |  |  |
| Male | 90 (78.9) | 30 (33.3) | 1.90 (0.65-5.59) | 0.238 |  | 5 (5.6) | 0.22 (0.059-0.85) | 0.052 |
| Female | 24 (21.1) | 5 (20.8) | 1 |  |  | 5 (20.8) | 1 |  |
| Antiviral prophylaxis^c^ |  |  |  |  |  |  |  |  |
| No | 29 (25.4) | 8 (27.6) | 0.69 (0.27-1.77) | 0.151 |  | 4 (13.8) | 1.78 (0.46-6.86) | 0.351 |
| Non-preferred drugs^d^ | 12 (10.5) | 1 (8.3) | 0.16 (0.020-1.35) |  |  | 0 (0) | 0.42 (0.022-7.85) |  |
| Preferred drugs^e^ | 73 (64.0) | 26 (35.6) | 1 |  |  | 6 (8.2) | 1 |  |
| ECOG performance status |  |  |  |  |  |  |  |  |
| ≤ 1 | 94 (82.5) | 31 (33.0) | 1.97 (0.61-6.39) | 0.253 |  | 10 (10.6) | 5.09 (0.29-90.56) | 0.275 |
| > 1 | 20 (17.5) | 4 (20.0) | 1 |  |  | 0 (0) | 1 |  |
| Cancer type |  |  |  |  |  |  |  |  |
| Hepatocellular carcinoma | 28 (24.6) | 13 (46.4) | 2.52 (1.04-6.12) | 0.038 |  | 4 (14.3) | 2.22 (0.58-8.53) | 0.422 |
| Others | 86 (75.4) | 22 (25.6) | 1 |  |  | 6 (7.0) | 1 |  |
| History of alcoholism |  |  |  |  |  |  |  |  |
| Yes | 17 (14.9) | 4 (23.5) | 0.66 (0.20-2.17) | 0.487 |  | 1 (5.9) | 0.61 (0.072-5.16) | 1.000 |
| No | 97 (85.1) | 31 (32.0) | 1 |  |  | 9 (9.3) | 1 |  |
| Liver involvement |  |  |  |  |  |  |  |  |
| Yes | 73 (64.0) | 25 (34.2) | 1.61 (0.68-3.82) | 0.274 |  | 8 (11.0) | 2.40 (0.48-11.88) | 0.449 |
| No | 41 (36.0) | 10 (24.4) | 1 |  |  | 2 (4.9) | 1 |  |
| Liver cirrhosis |  |  |  |  |  |  |  |  |
| Yes | 33 (28.9) | 14 (42.4) | 2.11 (0.90-4.93) | 0.083 |  | 3 (9.1) | 1.06 (0.26-4.36) | 1.000 |
| No | 81 (71.1) | 21 (25.9) | 1 |  |  | 7 (8.6) | 1 |  |
| HBeAg status |  |  |  |  |  |  |  |  |
| Seropositive | 10 (8.8) | 4 (40.0) | 1.60 (0.41-5.95) | 0.758 |  | 2 (20.0) | 3.00 (0.54-16.57) | 0.213 |
| Seronegative | 104 (91.2) | 31 (29.8) | 1 |  |  | 8 (7.7) | 1 |  |
| Baseline HBV DNA level |  |  |  |  |  |  |  |  |
| Detectable | 35 (30.7) | 15 (42.9) | 2.21 (0.96-5.12) | 0.061 |  | 4 (11.4) | 1.57 (0.41-5.95) | 0.758 |
| Undetectable | 79 (69.3) | 20 (25.3) | 1 |  |  | 6 (7.6) | 1 |  |
| Previous lines of therapy |  |  |  |  |  |  |  |  |
| < 2 | 71 (62.3) | 25 (35.2) | 1.79 (0.76-4.23) | 0.180 |  | 7 (9.9) | 1.46 (0.36-5.97) | 0.853 |
| ≥ 2 | 43 (37.7) | 10 (23.3) | 1 |  |  | 3 (7.0) | 1 |  |
| Treatment modality |  |  |  |  |  |  |  |  |
| Anti-PD-1 monotherapy | 82 (72.8) | 27 (32.5) | 1.39 (0.55-3.50) | 0.489 |  | 7 (8.4) | 0.86 (0.21-3.56) | 1.000 |
| Combination therapy | 31 (27.2) | 8 (25.8) | 1 |  |  | 3 (9.7) | 1 |  |
| Concurrent steroids^f^ |  |  |  |  |  |  |  |  |
| Yes | 14 (12.3) | 6 (42.9) | 1.85 (0.59-5.76) | 0.457 |  | 2 (14.3) | 1.92 (0.36-10.10) | 0.784 |
| No | 100 (87.7) | 29 (29.0) | 1 |  |  | 8 (8.0) | 1 |  |

^a^ Calculated using the χ2 test.

^b^ Calculated using the χ2 test except for HBeAg status and concurrent steroids, which were calculated using the Fisher exact test.

^c^ Antiviral drugs were classified as preferred drugs and non-preferred drugs, according to the 2018 American Association for the Study of Liver Diseases (AASLD) guideline.

^d^ Including lamivudine, adefovir and telbivudine.

^e^ Including entecavir and tenofovir.

^f^ Systemic steroids for any reason during immunotherapy (independent variable) including premedication, treatment for high intracranial pressure and treatment for irAEs (except immune-related hepatitis which is part of hepatitis [dependent variable] analyzed).

Abbreviations: HBV, hepatitis B virus; ECOG, Eastern Cooperative Oncology Group; HBeAg, hepatitis B e antigen; PD-1, programmed cell death protein-1; OR, odds ratio; CI, confidence interval.
