# Supplementary material for: Hepatitis B virus reactivation in cancer patients with positive Hepatitis B surface antigen undergoing PD-1 inhibition
Source: J Immunother Cancer. 2019 Nov 21;7:322. doi: 10.1186/s40425-019-0808-5 (PMC6873745; doi:10.1186/s40425-019-0808-5)
Supplement: Supplementary file 3 — Additional file 3: Table S3. Analysis of factors associated with immune-related hepatitis. [file 40425_2019_808_MOESM3_ESM.docx]

**Supplementary Table 3．Analysis of factors associated with immune-related hepatitis**

|  | No. of patients (%) | Any grade immune-related hepatitis | | |
| --- | --- | --- | --- | --- |
|  |  | No. of events (%) | OR (95% CI) | P value^a^ |
| Age, years |  |  |  |  |
| < 40 | 26 (22.8) | 4 (15.4) | 1.27 (0.37-4.39) | 0.958 |
| ≥ 40 | 88 (77.2) | 11 (12.5) | 1 |  |
| Gender |  |  |  |  |
| Male | 90 (78.9) | 13 (14.4) | 1.86 (0.39-8.86) | 0.655 |
| Female | 24 (21.1) | 2 (8.3) | 1 |  |
| Antiviral prophylaxis^b^ |  |  |  |  |
| No | 29 (25.4) | 2 (6.9) | 0.34 (0.072-1.62) | 0.123 |
| Non-preferred drugs^c^ | 12 (10.5) | 0 (0) | 0.18 (0.010-3.21) |  |
| Preferred drugs^d^ | 73 (64.0) | 13 (17.8) | 1 |  |
| ECOG performance status |  |  |  |  |
| ≤ 1 | 94 (82.5) | 15 (16.0) | 7.99 (0.46-139.27) | 0.120 |
| > 1 | 20 (17.5) | 0 (0) | 1 |  |
| Cancer type |  |  |  |  |
| Hepatocellular carcinoma | 28 (24.6) | 5 (17.9) | 1.65 (0.51-5.32) | 0.600 |
| Others | 86 (75.4) | 10 (11.6) | 1 |  |
| History of alcoholism |  |  |  |  |
| Yes | 17 (14.9) | 3 (17.6) | 1.52 (0.38-6.07) | 0.838 |
| No | 97 (85.1) | 12 (12.5) | 1 |  |
| Liver involvement |  |  |  |  |
| Yes | 73 (64.0) | 10 (13.7) | 1.14 (0.36-3.61) | 0.820 |
| No | 41 (36.0) | 5 (12.2) | 1 |  |
| Liver cirrhosis |  |  |  |  |
| Yes | 33 (28.9) | 4 (12.1) | 0.88 (0.26-2.98) | 1.000 |
| No | 81 (71.1) | 11 (13.6) | 1 |  |
| HBeAg status |  |  |  |  |
| Seropositive | 10 (8.8) | 2 (20.0) | 1.75 (0.33-9.16) | 0.857 |
| Seronegative | 104 (91.2) | 13 (12.5) | 1 |  |
| Baseline HBV DNA level |  |  |  |  |
| Detectable | 35 (30.7) | 6 (17.1) | 1.61 (0.53-4.93) | 0.591 |
| Undetectable | 79 (69.3) | 9 (11.4) | 1 |  |
| Previous lines of therapy |  |  |  |  |
| < 2 | 71 (62.3) | 12 (16.9) | 2.71 (0.72-10.23) | 0.129 |
| ≥ 2 | 43 (37.7) | 3 (7.0) | 1 |  |
| Treatment modality |  |  |  |  |
| Anti-PD-1 monotherapy | 82 (72.8) | 12 (14.5) | 0.63 (0.17-2.42) | 0.718 |
| Combination therapy | 31 (27.2) | 3 (9.7) | 1 |  |
| Concurrent steroids^e^ |  |  |  |  |
| Yes | 14 (12.3) | 4 (28.6) | 3.23 (0.87-12.09) | 0.162 |
| No | 100 (87.7) | 11 (11.0) | 1 |  |

^a^ Calculated using the χ2 test.

^b^ Antiviral drugs were classified as preferred drugs and non-preferred drugs, according to the 2018 American Association for the Study of Liver Diseases (AASLD) guideline.

^c^ Including lamivudine, adefovir and telbivudine.

^d^ Including entecavir and tenofovir.

^e^ Systemic steroids for any reason during immunotherapy (independent variable) including premedication, treatment for high intracranial pressure and treatment for irAEs (except immune-related hepatitis which is part of hepatitis [dependent variable] analyzed).

Abbreviations: HBV, hepatitis B virus; ECOG, Eastern Cooperative Oncology Group; HBeAg, hepatitis B e antigen; PD-1, programmed cell death protein-1; OR, odds ratio; CI, confidence interval.
